# Pan-Immune-Inflammatory Value (PIV) and HALP Score as Independent Prognostic Indicators in Acute Coronary Syndrome Patients

**DOI:** 10.3390/jcm15041660

**Published:** 2026-02-22

**Authors:** Azmi Eyiol, Hatice Eyiol, Ahmet Yilmaz, Hasan Sari

**Affiliations:** 1 Department of Cardiology, Beyhekim Training and Research Hospital, 42100 Konya, Turkey; 2 Department of Anesthesiology and Reanimation, Beyhekim Training and Research Hospital, 42100 Konya, Turkey; hatice.eyiol@saglik.gov.tr; 3 Department of Cardiology, Faculty of Medicine, Karamanoğlu Mehmetbey University, 70100 Karaman, Turkey; drahmetyilmaz@kmu.edu.tr; 4 Department of Cardiology, Kemer State Hospital, 07100 Antalya, Turkey; hasan.sari2@saglik.gov.tr

**Keywords:** acute coronary syndrome, Pan-Immune-Inflammatory Value, HALP score, prognostic marker, systemic inflammation

## Abstract

**Introduction:** Acute coronary syndrome (ACS), encompassing unstable angina, NSTEMI, and STEMI, is a major cause of morbidity and mortality worldwide. Novel inflammatory and nutritional biomarkers may provide incremental value for risk stratification beyond conventional predictors. This work sought to determine whether the Pan-Immune-Inflammatory Value (PIV) and the Hemoglobin-Albumin-Lymphocyte-Platelet (HALP) score could serve as independent prognostic indicators in individuals presenting with acute coronary syndrome. **Methods:** A retrospective multicenter study included ACS patients hospitalized between January 2020 and May 2024. Demographics, clinical data, and laboratory results were collected. PIV was calculated as follows: neutrophils × platelets × monocytes/lymphocytes. HALP score was calculated as follows: hemoglobin × albumin × lymphocytes/platelets. Correlations with clinical parameters and mortality prediction were analyzed. **Results:** A total of 1134 patients (mean age 62 ± 12 years) were included. PIV showed positive correlations with WBC (Rho = 0.574), troponin (Rho = 0.381), and CRP (Rho = 0.295), and negative correlations with HDL (Rho = –0.101) and ejection fraction (Rho = –0.316) (all *p* < 0.01). PIV independently predicted mortality with a cut-off ≥1074.2 (AUC = 0.619, sensitivity 45%, specificity 79.9%). HALP score negatively correlated with age, troponin, CRP, and ICU stay, and predicted mortality with a cut-off ≤3.58 (AUC = 0.722, sensitivity 53.8%, specificity 82%). Comparative ROC analysis showed that HALP demonstrated superior discriminative ability for mortality prediction compared with PIV. **Conclusions:** PIV and HALP score are independent prognostic markers in ACS, reflecting inflammatory burden and nutritional status. Their integration into clinical workflows may enhance risk stratification and support individualized management strategies. Given their simplicity and universal availability, PIV and HALP may serve as practical adjunctive tools to established risk scores, enabling early identification of high-risk ACS patients at the time of admission.

## 1. Introduction

Acute coronary syndrome (ACS) refers to a spectrum of clinical conditions caused by abrupt reductions in coronary blood flow, which includes unstable angina, non-ST-elevation myocardial infarction (NSTEMI), and ST-elevation myocardial infarction (STEMI) [1,2]. Collectively, these disorders represent one of the leading causes of morbidity and mortality worldwide, emphasizing the ongoing need to identify novel biomarkers that can improve prognostic accuracy and guide therapeutic decision-making [3]. In this context, particular attention has been directed toward markers of inflammation and immune activity, given their central role in the pathophysiology of ACS [4].

The pan-immune-inflammatory value (PIV) is a recently proposed composite index that integrates multiple hematological indicators of systemic inflammation [5]. Preliminary studies suggest that PIV reflects the overall immune–inflammatory balance of the patient and may serve as a surrogate of disease severity and outcomes in various clinical scenarios. Nevertheless, its prognostic role in ACS remains insufficiently clarified.

While many individual inflammatory biomarkers have been associated with adverse outcomes in ACS, a global index such as PIV could provide a more comprehensive assessment [6]. Correlating PIV with conventional clinical and biochemical measures—including white blood cell count (WBC), troponin, C-reactive protein (CRP), and fibrinogen—may improve risk stratification and support more personalized management strategies [7]. On this basis, this study sought to explore the relationship between PIV and various clinical as well as laboratory parameters in individuals diagnosed with ACS, as well as to determine its predictive value for mortality. In addition, subgroup analyses according to demographic and clinical features were conducted to evaluate whether PIV may be applied as a practical prognostic marker in daily practice.

Inflammation in ACS is also closely linked to hematological and nutritional factors. Lymphocyte depletion, along with increased platelet and neutrophil counts, reflects heightened immune activation, whereas alterations in hemoglobin concentration and serum albumin are established predictors of poor cardiovascular outcomes [8]. The hemoglobin, albumin, lymphocyte, and platelet (HALP) score, which incorporates these variables, has been increasingly recognized as a measure of both systemic inflammation and nutritional status [9]. Beyond individual parameters, composite indices integrating hematological and nutritional components have been proposed to better reflect the systemic inflammatory milieu and overall physiological reserve in cardiovascular diseases [10]. Recent evidence indicates that a low HALP score is associated with higher mortality among patients with coronary artery disease (CAD). Similarly, PIV has been linked to disease burden and prognosis in CAD populations [11,12]. However, limited data exist regarding the combined prognostic significance of these indices in ACS. Accordingly, the current investigation aimed to explore the prognostic roles of PIV and HALP score in ACS, focusing on their relationships with clinical outcomes and recovery patterns [13]. To our knowledge, this is the first large multicenter study simultaneously evaluating PIV and HALP in ACS across clinical severity strata.

## 2. Methods

### 2.1. Study Design

This investigation was designed as a retrospective, multicenter study to assess the prognostic impact of the PIV and the HALP score in patients with a confirmed diagnosis of ACS. This multicenter retrospective study was conducted at two tertiary institutions in Konya, Türkiye: Konya Beyhekim Training and Research Hospital and Necmettin Erbakan University Meram Faculty of Medicine Hospital. Data were retrieved from the electronic medical record systems of two hospitals over a five-year period, spanning 1 January 2020, to 1 May 2024. A total of 1134 individuals were included, of whom 811 presented with STEMI and 323 with NSTEMI and USAP. Patients with missing baseline data required for index calculation and multivariable analyses were excluded (*n* = 173). The protocol received approval from the institutional ethics committee (decision number 4999, dated 7 June 2024), and all procedures were conducted in accordance with the ethical principles outlined in the Declaration of Helsinki. Due to the retrospective nature of the study, the requirement for informed consent was waived by the ethics committee. Identifiable patient information was handled in compliance with ethical standards, ensuring confidentiality throughout the study process.

### 2.2. Patient Evaluation

Eligible participants were adults (≥18 years) with a confirmed diagnosis of ACS, including unstable angina, NSTEMI, and STEMI, verified through clinical presentation, electrocardiographic findings, and cardiac biomarkers. The study population included both ST-segment elevation myocardial infarction (STEMI) and non–ST-segment elevation myocardial infarction (NSTEMI) patients. The diagnosis and classification of ACS subtypes were established according to the most recent guideline-based criteria and the universal definition of myocardial infarction. Only patients with complete clinical, laboratory, and follow-up information were included. Exclusion criteria comprised the presence of active infection, autoimmune disorders, malignancy, or recent use of immunosuppressive therapy within the preceding six months. Patients with missing in-hospital outcome status were excluded.

Comprehensive clinical information was extracted from institutional electronic health records. The dataset included demographic variables (age, sex), presenting symptoms, laboratory analyses, imaging results, and therapeutic outcomes. Key laboratory and clinical indicators recorded were white blood cell (WBC) count, hemoglobin, neutrophils, monocytes, lymphocytes, platelet (PLT), red cell distribution width (RDW), serum albumin, lipid profile [low-density lipoprotein (LDL), high-density lipoprotein (HDL), triglycerides], left ventricular ejection fraction (EF), SYNTAX score, TIMI risk score, troponin, CRP, ferritin, D-dimer, fibrinogen, HEART score, blood glucose, uric acid, as well as length of stay in both the intensive care unit (ICU) and the hospital. All laboratory parameters used for PIV and HALP calculation were obtained from the first blood samples collected at emergency department admission.

To quantify systemic inflammation, the PIV was calculated using the formula:

PIV = (neutrophil count × platelet count × monocyte count)/lymphocyte count.

This composite index reflects the interplay of immune and inflammatory responses. Nutritional and inflammatory status was further evaluated using the HALP score, calculated as:

HALP = hemoglobin × albumin × lymphocyte count/platelet count.

The primary endpoint of the study was in-hospital all-cause mortality, defined as death occurring during the index hospitalization. Adjudicated cardiovascular mortality and detailed MACE components were not consistently available across all centers due to the retrospective multicenter nature of the dataset.

To minimize confounding, multivariable models were constructed using baseline demographic and clinical variables available consistently across both participating centers. Although key clinical severity markers such as LVEF and troponin were available and included in the multivariable models, detailed information on revascularization status (PCI/CABG) and discharge medication use was not consistently recorded across the retrospective multicenter dataset and therefore could not be incorporated into the adjusted analyses.

### 2.3. Statistical Analysis

All statistical procedures were carried out using SPSS version 31.0 (IBM Corp., Chicago, IL, USA) and R. The distribution of continuous variables was assessed through the Kolmogorov–Smirnov test, inspection of histograms, skewness/kurtosis values, and Q–Q plots. Categorical data were summarized as absolute numbers (N) and percentages (%). For quantitative variables, results were presented either as mean ± standard deviation for normally distributed data or as median with interquartile range (IQR; minimum–maximum) for non-normally distributed data. Comparisons between two independent groups were conducted using the independent samples *t*-test when normality assumptions were met, and the Mann–Whitney U test otherwise. For analyses involving more than two groups, the Kruskal–Wallis H test was applied, followed by Bonferroni-adjusted post hoc comparisons. Homogeneity of variances was checked using the Levene test. To evaluate factors associated with binary outcomes, univariate and multivariate logistic regression analyses were performed. Receiver operating characteristic (ROC) curve analysis was used to determine cut-off values and predictive performance of key parameters. Correlation analyses between continuous variables were assessed using Spearman’s rank correlation coefficients. Multivariable logistic regression models were constructed to evaluate the independent association of PIV and HALP with in-hospital all-cause mortality. Results are presented as adjusted odds ratios (ORs) with 95% confidence intervals (CIs). ROC analyses were performed to determine optimal cut-off values of PIV and HALP for predicting in-hospital mortality.

To explore potential non-linear associations between PIV/HALP and in-hospital mortality, restricted cubic spline (RCS) analyses were additionally performed within the multivariable logistic regression framework. Spline models were constructed using 3–5 knots placed at prespecified percentiles of the distribution, and results were presented as adjusted odds ratios with the median value of each biomarker as the reference. Evidence of non-linearity was assessed by comparing the spline model with a linear term model. Spline-based dose–response curves were visualized accordingly.

Multivariable logistic regression models were constructed using baseline covariates selected a priori based on clinical relevance and established ACS prognostic literature. Troponin and CRP were log-transformed due to skewed distributions. Collinearity was assessed using variance inflation factors (VIF), and variables with high collinearity were not simultaneously included in the same model.

All statistical tests were two-sided, with a significance threshold set at *p* < 0.05.

## 3. Results

A total of 1134 patients hospitalized with acute coronary syndrome between January 2020 and May 2024 were included in the final analysis. The median length of hospital stay was 5 days (IQR: 3–5), with a mean duration of 4.6 ± 2.6 days. Table 1 summarizes the distribution of quantitative parameters in patients with ACS. The patients’ ages ranged from 31 to 94 years, with a mean age of 62 ± 12 years. Hemoglobin levels varied from 4.0 to 19 g/dL, averaging 14.1 ± 1.9 g/dL. WBC counts showed a wide range, with a median of 9.71 × 10^3^/μL (1.9–35.8), while neutrophil counts had a median of 6.9 × 10^3^/μL (1.3–28.8). PLT spanned from 2.9 to 909 × 10^3^/μL, with a median of 240 × 10^3^/μL. Troponin concentrations demonstrated wide variability, spanning from 2 to 48,000 ng/L, with a median value of 7800 ng/L. D-dimer levels showed a median of 890 ng/mL (range: 220–3510), while fibrinogen levels averaged 3.9 g/L (range: 2.65–4.98).

Males had a lower mean age (60.82 ± 11.84) compared to females (65.98 ± 11.08) with a significant difference (*p* < 0.001). Hemoglobin concentrations were markedly higher in men (14.45 ± 1.82 g/dL) than in women (12.56 ± 1.47 g/dL; *p* < 0.001). Male subjects also demonstrated greater WBC and neutrophil counts, with median values of 9.93 (1.9–35.8) × 10^3^/μL and 7.07 (1.3–28.8) × 10^3^/μL, respectively. Females exhibited elevated platelet counts, with a median of 262.5 (3.7–501) × 10^3^/μL. By comparison, males had notably higher troponin levels (median 8000 ng/L) relative to females (median 4323 ng/L; *p* < 0.001). D-dimer levels were slightly higher in males with a median of 891 ng/mL compared to 880.5 ng/mL in females (*p* = 0.02).

Table 2 shows a comparison of PIVs in ACS patients according to the presence of specific conditions. PIV values were significantly higher in patients receiving inotropic support and those receiving mechanical respiratory support. PIV values were higher in patients who were exitus than in those who were discharged. The highest PIV values were found in those diagnosed with STEMI, and higher in those subsequently diagnosed with NSTEMI than in those diagnosed with USAP. The highest PIV rate was found in those with TIMI flow of zero and those with KILLIP classification 4 (*p* < 0.001). Table 3 shows a summary of the correlation relationships of the PIV parameter with other quantitative parameters in ACS patients. The PIV value shows a moderately positive correlation with WBC.

Table 4 shows comparison of HALP score in ACS patients according to the presence of specific conditions. HALP score were significantly lower in patients receiving inotropic support and those receiving mechanical respiratory support (*p* < 0.001). HALP score were lower in patients who were exitus than in those who were discharged (*p* < 0.001). The lowest HALP score were found in those diagnosed with STEMI, and higher in those subsequently diagnosed with NSTEMI than in those diagnosed with USAP (*p* = 0.003). The lowest HALP score was found in those with TIMI flow of zero and those with KILLIP classification 4 (*p* < 0.001). Table 5 shows summary of correlation relationships of HALP score with other quantitative parameters in ACS patients.

In multivariable logistic regression analysis, both PIV and HALP remained independently associated with in-hospital all-cause mortality after adjustment for relevant confounders. Univariate and multivariate logistic regression analyses for mortality in ACS patients are summarized in Table 6 and Table 7. Both tables show a significant correlation with PIV and HALP scores (*p* < 0.001). Table 8 summarizes the ROC analysis of quantitative parameters for mortality in ACS patients, predictive values, and cut-off values. The highest AUC value was for the TIMI score: 0.867 (0.831–0.903), with a cut-off value of ≥5.5 (*p* < 0.001), with a sensitivity of 93.8% and a specificity of 75.9%. The AUC value of the HALP score: 0.722 (0.656–0.788), with a cut-off value of ≤3.58 (*p* < 0.001), with a sensitivity of 53.8% and a specificity of 82%. The PIV: 0.619 (0.551–0.687), with a cut-off value of ≥1074.2 (*p* = 0.001), with a sensitivity of 45% and a specificity of 79.9%.

**Figure 1:**
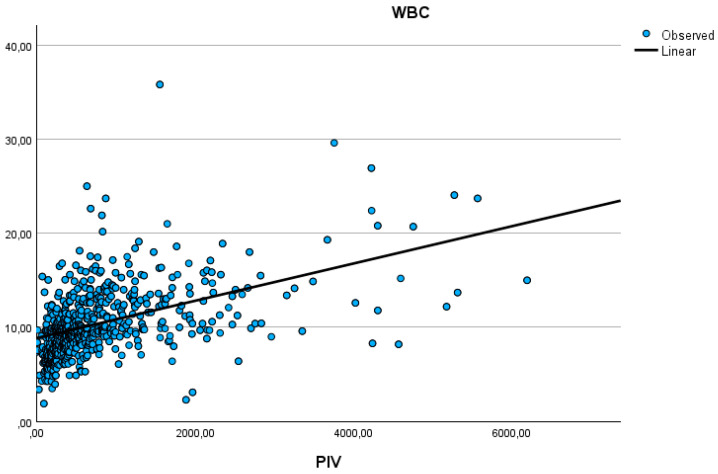
The moderate positive correlation relationship between PIV and WBC (rho = 0.574, *p* < 0.001).

**Figure 2:**
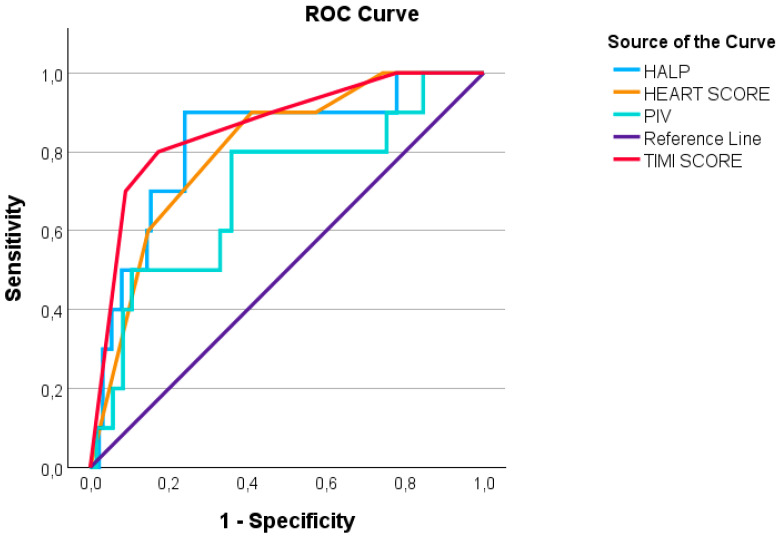
ROC analysis graph in terms of mortality for significant predictive variables.

Figure 1 shows the moderate positive correlation relationship between PIV and WBC (rho = 0.574, *p* < 0.001). Figure 2 shows the ROC analysis graph in terms of mortality for significant predictive variables.

**Figure 3:**
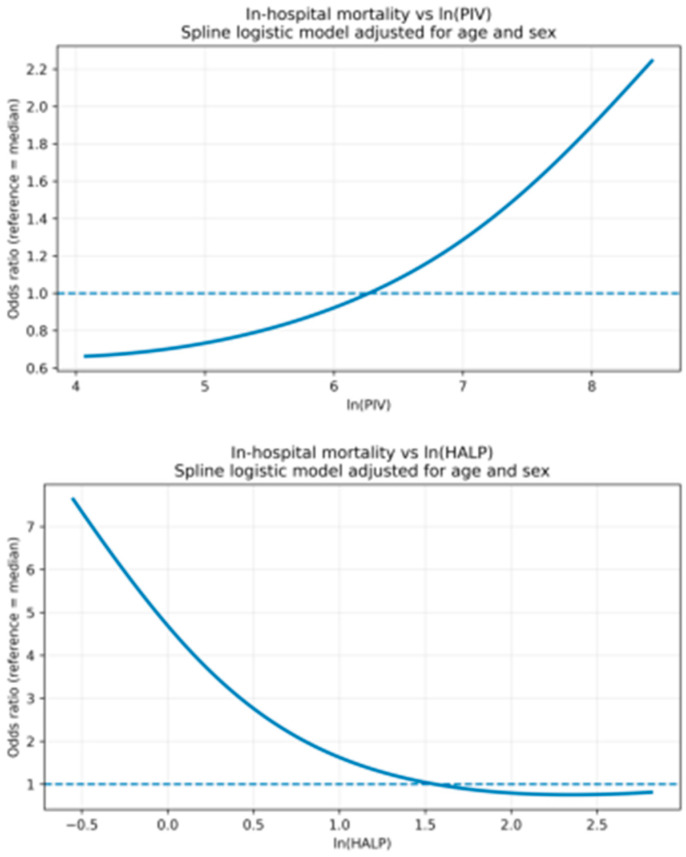
Restricted cubic spline analysis of PIV and HALP for in-hospital mortality. Restricted cubic spline curves illustrate the association between ln(PIV) (Upper panel) and ln(HALP) (Bottom panel) and the risk of in-hospital mortality in patients with acute coronary syndrome. Models were fitted using spline-based multivariable logistic regression and were adjusted for age and sex. The y-axis represents the odds ratio (OR) relative to the median value of each biomarker (reference OR = 1, dashed line).

Restricted cubic spline analyses based on multivariable logistic regression demonstrated that the associations between ln(PIV) and ln(HALP) with in-hospital all-cause mortality remained robust after adjustment for clinically relevant covariates, including age, sex, LVEF, TIMI score, Killip class, ACS subtype (USAP/NSTEMI/STEMI), and major comorbidities (diabetes mellitus, hypertension, and hyperlipidemia), as well as inflammatory markers (log-transformed troponin and CRP). In spline models, higher ln(PIV) values were associated with progressively increased mortality risk, whereas higher ln(HALP) values showed a consistent inverse relationship with in-hospital mortality (Figure 3).

In incremental model analyses, the TIMI score alone demonstrated strong discrimination for in-hospital mortality (AUC = 0.867). Adding HALP increased the AUC to 0.903 and yielded higher reclassification performance (continuous NRI = 0.646; IDI = 0.068), whereas PIV provided only a modest improvement (AUC = 0.880; continuous NRI = 0.194; IDI = 0.010) (Table 9).

## 4. Discussion

ACS contributes significantly to morbidity and mortality worldwide, and ongoing research is needed to identify reliable biomarkers for prognosis and treatment strategies. It is important to investigate the prognostic significance of the Pan-Immune-Inflammatory Value (PIV) and HALP score in ACS patients and to evaluate their correlation with clinical and laboratory parameters.

The PIV has emerged as a significant biomarker in evaluating the prognosis of patients with ACS [14]. In our study, PIV showed a significant positive correlation with several important inflammatory and cardiac damage markers, including WBC, troponin, and CRP. This aligns with existing literature that underscores the importance of systemic inflammation in the pathophysiology of ACS. Elevated levels of these markers in conjunction with higher PIV suggest a state of heightened inflammatory response, which has been linked to adverse cardiovascular events [13].

Inflammatory and coagulative pathways are tightly linked, each amplifying the activity of the other. Among immune cells, neutrophils have a pivotal function in both atherogenesis and thrombus development. Whereas elevated neutrophil counts indicate sustained inflammatory activity, lymphocyte numbers more accurately reflect adaptive immune control mechanisms [13]. During acute inflammatory responses, neutrophils, monocytes, and platelets tend to rise, while lymphocyte levels usually decline [14]. This lymphopenic response has been consistently associated with unfavorable cardiovascular outcomes [15,16]. Moreover, platelets act not only as mediators of thrombosis but also as active participants in both the early and long-term inflammatory cascades of coronary artery disease [11].

Our findings revealed that PIV positively correlates with WBC (Rho = 0.574, *p* < 0.001), troponin (Rho = 0.381, *p* < 0.001), and CRP (Rho = 0.295, *p* < 0.001), suggesting that individuals with elevated PIV scores are more likely to have more severe inflammatory and cardiac injury profiles. Similar observations have been reported in previous investigations, particularly in STEMI patients undergoing primary PCI and in high-risk ACS cohorts [15,16,17]. The strong association between PIV and these markers highlights its potential as a comprehensive measure of inflammatory burden in ACS.

The inverse correlation between PIV and HDL cholesterol (Rho = −0.101, *p* = 0.001), as well as EF (Rho = −0.316, *p* < 0.001), further supports the role of PIV in reflecting the overall health status of ACS patients. Lower HDL levels and reduced EF are indicative of poorer cardiovascular health, and their association with higher PIV scores suggests that elevated inflammatory and nutritional risk indices are detrimental to lipid metabolism and cardiac function. These findings are in line with studies that have reported similar relationships between inflammatory markers, lipid profiles, and cardiac performance [18].

Moreover, PIV was identified as a moderate predictor of mortality in ACS patients, with an optimal cut-off value of ≥1074.2, providing a sensitivity of 45% and specificity of 79.9%. This finding is supported by prior research indicating that higher PIV is associated with increased mortality risk in cardiac patients [19,20]. The ROC analysis for PIV showed modest discrimination for mortality (AUC 0.619) with limited sensitivity at the proposed cut-off, suggesting that PIV should be interpreted as an adjunct marker rather than a stand-alone risk tool.

The TIMI score, a well-established tool for assessing ACS severity, demonstrated the strongest association with mortality in our cohort, with an odds ratio of 4.055 and 5.129 in univariate and multivariate logistic regression analyses, respectively. This is consistent with the pioneering study by Antman et al. demonstrating the prognostic utility of the TIMI score in predicting adverse outcomes in ACS patients [21]. Combining PIV with the TIMI score may improve the accuracy of risk stratification, allowing for more targeted therapeutic interventions.

Age was also a significant predictor of mortality, with older patients exhibiting higher odds of adverse outcomes. This is consistent with extensive literature indicating that advanced age is a critical risk factor for increased mortality and complications in ACS [22]. The higher vulnerability of elderly patients to systemic inflammation and comorbidities likely contributes to this increased risk [23]. Therefore, incorporating age-specific considerations into the management of ACS is essential for improving outcomes.

Interestingly, PIV did not show a significant correlation with the SYNTAX score, which evaluates the anatomical complexity of coronary artery disease. This suggests that while PIV captures the inflammatory and nutritional dimensions of ACS, it does not reflect the anatomical severity of coronary lesions. The inflammatory response during acute coronary syndrome may vary, therefore the relationship may not be clearly established. A large-scale study in patients with stable angina pectoris may provide a more accurate answer. This observation is also suggested by prior studies emphasizing the dissociation between inflammatory burden and anatomical complexity in coronary artery disease [18]. Consequently, a multidimensional approach incorporating both anatomical and inflammatory assessments is crucial for a holistic evaluation of ACS patients.

Recent studies suggest that the HALP score may also be used to predict mortality in individuals with cardiovascular disease [24,25]. According to Zheng et al., patients with a HALP score ≤ 69.68 had a higher risk of all-cause mortality. Furthermore, the HALP score had a sensitivity of 0.510, a specificity of 0.654, and an AUC of 0.610 for predicting cardiovascular disease prognosis, outperforming albumin, lymphocyte, and platelet levels alone [11]. Karakayalı and colleagues analyzed a cohort of individuals with coronary artery disease and demonstrated that the HALP index served as an independent determinant of in-hospital mortality, based on a Cox regression model [12]. In addition, a recent observational cohort study reported that both PIV and HALP score were independently associated with disease prognosis and recovery in patients with acute pericarditis, supporting the broader applicability of these indices across inflammatory cardiac disorders [26]. In a separate investigation from our group that explored the prognostic implications of PIV and HALP indices in critically ill patients both with and without atrial fibrillation each marker showed a significant association with the presence of AF. However, although the HALP index exhibited a strong and consistent prognostic performance, the mortality-predictive capacity of PIV appeared comparatively limited [27]. 

In our study, lower HALP scores were significantly associated with older age (Rho = −0.291, *p* < 0.001), higher troponin levels (Rho = −0.200, *p* < 0.001), increased CRP (Rho = −0.231, *p* < 0.001), and longer ICU stay (Rho = −0.143, *p* < 0.001). This suggests that patients with lower HALP scores have more severe inflammatory and cardiac injury profiles and a greater propensity for ICU stays. The relationship between the HALP score and these markers highlights its potential as a comprehensive measure of inflammatory burden in ACS.

The positive correlation between the HALP score and EF (Rho = 0.2, *p* < 0.001) further supports the role of the HALP score in reflecting the overall health status of ACS patients. Decreased EF is a marker of poorer cardiovascular health, and its association with a lower HALP score suggests that high inflammatory indices have a negative impact on cardiac function. These findings are consistent with studies reporting similar associations between inflammatory markers and cardiac performance [24,25].

Furthermore, the HALP score has been identified as a moderate predictor of mortality in ACS patients, with an optimal cut-off value of ≤3.58, providing 53.8% sensitivity and 82% specificity. In our study, ROC analysis for HALP showed a remarkable AUC of 0.722, highlighting its prognostic value. Therefore, we suggest that the HALP score should be used in conjunction with other clinical parameters for comprehensive risk assessment.

Another important point to consider is that patients with a high PIV ratio and a low HALP score require more inotropic support and mechanical ventilation, and their exitus rates are significantly higher. Similarly, this ratio is significant in the STEMI group of ACS patients, and this significance persists even in cases where the Killip classification is 4 and the TIMI flow is 0. This suggests that a high PIV ratio and a low HALP score provide important information regarding patient prognosis, need for support, potential adverse events following coronary intervention, discharge planning, and exitus situations.

In addition to inflammation- and nutrition-based composite indices, other recently proposed risk models and glucometabolic indices have also demonstrated prognostic value in ACS populations. These emerging tools highlight the multifactorial nature of adverse outcomes after ACS and suggest that integrated models incorporating metabolic, inflammatory, and clinical severity domains may provide the highest prognostic accuracy [28].

Beyond circulating biomarkers, non-invasive imaging plays an important role in ACS prognostication. Cardiac magnetic resonance (CMR), particularly late gadolinium enhancement (LGE), provides robust information regarding myocardial scar burden, infarct size, and viability, which are strongly associated with clinical outcomes. Therefore, systemic inflammatory and nutritional indices such as PIV and HALP may be considered complementary tools, especially in real-world settings where advanced imaging modalities are not routinely available or feasible [29].

From a methodological standpoint, both PIV and HALP indices can be derived from standard laboratory measurements that are routinely performed in everyday clinical practice across the globe. Owing to their simplicity and universal availability, these composite markers may offer meaningful information regarding disease prognosis and overall clinical condition in patients with acute coronary syndrome. Nevertheless, additional prospective investigations are warranted to validate their clinical applicability. Future studies should also explore the potential impact of demographic background, coexisting illnesses, and the length of clinical follow-up on the predictive performance of these indices.

## 5. Limitations

This study has several limitations. First, its retrospective observational design is inherently subject to selection bias and residual confounding. Second, although the analysis was adjusted for clinically relevant covariates, unmeasured confounders and center-level heterogeneity cannot be fully excluded. Third, the absence of an external validation cohort limits the generalizability of our findings. Therefore, prospective studies with standardized data collection and external validation in independent cohorts are warranted. Additionally differences in clinical practices and documentation procedures across participating centers could have influenced the uniformity of the dataset. Moreover, not all possible confounding factors were controlled for, which may have affected the observed associations. Finally, the absence of extended follow-up restricts the ability to evaluate long-term prognostic implications of PIV, HALP score, and other biomarkers in patients with ACS. Residual confounding cannot be excluded despite multivariate adjustment. It should be acknowledged that STEMI and NSTEMI represent clinically heterogeneous ACS phenotypes. These entities may differ in baseline risk, ischemic burden, treatment strategies, and inflammatory response, which may influence PIV and HALP values as well as mortality risk. Therefore, despite multivariable adjustment, residual confounding related to STEMI/NSTEMI heterogeneity cannot be fully excluded, and this should be considered when interpreting the prognostic associations observed in the present retrospective analysis. Furthermore, the absence of an external validation cohort restricts the generalizability of our findings and warrants confirmation in future prospective studies.

## 6. Conclusions

Our study highlights the prognostic significance of PIV and HALP score in ACS patients, emphasizing its role in reflecting systemic inflammation and nutritional status. These findings suggest that integrating PIV and HALP score into clinical practice could improve risk stratification and guide personalized treatment strategies. PIV and HALP may serve as practical inflammatory–nutritional indices for in-hospital risk stratification in ACS; however, prospective validation in independent cohorts is required before clinical implementation.

## Figures and Tables

**Table 1:** Baseline distribution of quantitative clinical and laboratory parameters in ACS.

Parameters	Units	Minimum	Maximum	Distribution
Age	years	31	94	62.0 ± 12.0
Hemoglobin	g/dL	4	19	14.1 ± 1.9
White Blood Cell	10^3^/μL	2	36	9.71 (1.9–35.8)
Neutrophil	10^3^/μL	1.3	28.8	6.9 (1.3–28.8)
Monocyte	10^3^/μL	0.03	1.78	0.63 (0.03–1.78)
Lymphocyte	10^3^/μL	0.2	8.40	2 (0.2–8.4)
Platelet	10^3^/μL	2.90	909.0	240 (2.9–909)
RDW	%	9.5	35.3	14 (9.5–35.3)
Albumin	g/L	14	51	40.6 (14.3–50.6)
LDL	mg/dL	33	243	162 (33–243)
HDL	mg/dL	12	80	37 (12.1–80)
Triglyceride	mg/dL	44.9	907.0	219 (44.9–907)
Ejection fraction	%	15	65	48 (15–65)
Syntax Score	-	1	50.5	10.8 (1–50.5)
TIMI score	-	1	7	5 (1–7)
Troponin	ng/L	2	48,000	7800 (2–48,000)
C Reactive Protein	mg/L	0.2	396	11 (0.2–396.0)
D-dimer	ng/mL	220	3510	890 (220–3510)
Ferritin	μg/L	13.0	198.0	86 (13–198)
Fibrinogen	g/L	2.65	4.98	3.9 ± 0.33
Heart Score	-	4	10	8 (4–10)
Glucose	mg/dL	88	340	111 (88–340)
Uric acid	mg/dL	5.4	11.8	6.5 (5.4–11.8)
PIV	-	1.83	6188.17	528.61 (1.83–6188.17)
HALP score	-	0.12	446.69	4.73 (0.12–446.69)
ICU Time	days	1	28	2 (1–28)
Hospitalization Time	days	0	28	5 (0–28)

Parameters are expressed as mean ± standard deviation or IQR (Interquartile Range) [median, min and max]. Abbreviations: ACS: Acute Coronary Syndrome; RDW: Red Cell Distribution Width; LDL: Low Density Lipoprotein; HDL: High Density Lipoprotein; PIV: Pan-immun-inflammatory value; ICU: Intensive Care Unit.

**Table 2:** Distribution of PIVs across subgroups of ACS patients with specific clinical conditions.

		PIV	*p*
Parameters		Median (Min–Max)	
Inotrope Support	No	514.04 (1.83–6188)	<0.001 *
	Yes	965.01 (74.38–5563.32)	
Mechanical Ventilation Support	No	514.16 (1.83–6188)	<0.001 *
	Yes	965.01 (74.38–5563.32)	
Discharge	Exitus	681.98 (74.38–5563.32)	<0.001 *
	Yes	514.16 (1.83–6188)	
Diagnosis	USAP	260.06 (94.62–1071.98) ^a^	<0.001 **
	NSTEMI	338.51 (1.83–4592.19) ^b^	
	STEMI	638.55 (4.04–6188) ^c^	
TIMI Flow	0	1222.16 (171.76–2531.17) ^a^	<0.001 **
	1	631.59 (87.85–5563.32) ^a,b^	
	2	252.99 (74.38–1317.12) ^c^	
	3	524.57 (1.83–6188) ^d^	
Killip classification (in STEMI patients)	Class I	617.88 (4.04–6188) ^a^	0.008 **
	Class II	756 (24.75–5171.46) ^a,b^	
	Class III	631.55 (87.85–4566.38) ^a,b^	
	Class IV	966.8 (74.38–5563.32) ^b^	
Hypertension	No	562.26 (1.83–5269.47)	0.011 *
	Yes	492.66 (4.04–6188)	
Hyperlipidemia	No	657.81 (4.04–5310.47)	0.003 *
	Yes	493.58 (1.83–6188)	
Diabetes Mellitus	No	560.44 (1.83–6188)	0.007 *
	Yes	450.17 (6.78–4750.43)	
Smoking	No	437.95 (57.97–5563.32)	0.127 *
	Yes	538.2 (1.83–6188)	
Obesity	No	533.64 (6.78–5563.32)	0.201 *
	Yes	510.7 (1.83–6188)	
Family History	No	509.75 (35.4–4750.43)	0.408 *
	Yes	528.61 (1.83–6188)	

* Mann–Whitney U test. ** Kruskal–Wallis-H test. The groups with significant differences are labeled with letters (a) (b) (c) (d). While there is no significant difference between the groups with the same letter label, there is a significant difference (*p* < 0.05) between the groups with different letter labels. Bold values indicate statistically significant results (*p* < 0.05). Abbreviations: ACS: Acute Coronary Syndrome; PIV: Pan-immun-inflammatory value.

**Table 3:** Summary of correlation relationships of PIV parameter with other quantitative parameters in ACS patients.

Parameters	PIV	
Age	Rho	0.033
	*p*	0.263
WBC	Rho	0.574
	*p*	**<0.001**
HDL	Rho	−0.101
	*p*	**0.001**
EF (%)	Rho	−0.316
	*p*	**<0.001**
SYNTAX Score	Rho	−0.015
	*p*	0.625
TIMI Score	Rho	0.157
	*p*	**<0.001**
Troponin	Rho	0.381
	*p*	**<0.001**
CRP	Rho	0.295
	*p*	**<0.001**
D-dimer	Rho	0.188
	*p*	**<0.001**
Ferritin	Rho	0.359
	*p*	**<0.001**
Fibrinogen	Rho	0.178
	*p*	**<0.001**
HEART score	Rho	0.163
	*p*	**0.003**
ICU time (day)	Rho	0.322
	*p*	**<0.001**
Hospitalization time (day)	Rho	0.256
	*p*	**<0.001**

Spearman correlation analysis (correlation coefficient = rho). Bold values indicate statistically significant results (*p* < 0.05). Abbreviations: ACS: Acute Coronary Syndrome; WBC: White Blood Cell; EF: Ejection Fraction; CRP: C Reactive Protein; HDL: High Density Lipoprotein; PIV: Pan-immun-inflammatory value; ICU: Intensive Care Unit.

**Table 4:** Comparison of HALP score in ACS patients according to the presence of specific conditions.

		HALP Score	*p*
Parameters		Median (Min–Max)	
Inotrope Support	No	4.86 (0.42–446.69)	<0.001 *
	Yes	2.35 (0.12–9.68)	
Mechanical Ventilation Support	No	4.84 (0.42–446.69)	<0.001 *
	Yes	2.48 (0.12–9.68)	
Discharge	Exitus	2.61 (0.12–9.68)	<0.001 *
	Yes	4.86 (0.42–446.69)	
Diagnosis	USAP	5.43 (1.39–14.21) ^a^	0.003 **
	NSTEMI	4.8 (0.6–384.5) ^a,b^	
	STEMI	4.69 (0.12–446.69) ^b^	
TIMI Flow	0	1.49 (0.13–7.49) ^a^	<0.001 **
	1	2.83 (0.12–9.68) ^a,b^	
	2	7.32 (1.83–12.9) ^c^	
	3	4.83 (0.42–446.69) ^c,d^	
Killip classification (in STEMI patients)	Class I	5.05 (0.42–446.69)^a^	<0.001 **
	Class II	4.14 (0.74–13.86) ^b^	
	Class III	3.17 (0.52–12.03) ^b^	
	Class IV	2.07 (0.12–9.68) ^b^	
Hypertension	No	5.54 (0.66–384.5)	<0.001 *
	Yes	4.57 (0.12–446.69)	
Hyperlipidemia	No	4.3 (0.13–446.69)	0.004 *
	Yes	4.86 (0.12–384.5)	
Diabetes Mellitus	No	4.78 (0.13–446.69)	0.265 *
	Yes	4.66 (0.12–30.32)	
Smoking	No	3.79 (0.42–13.87)	<0.001 *
	Yes	5.08 (0.12–446.69)	
Obesity	No	4.66 (0.12–30.32)	0.690 *
	Yes	4.8 (0.13–446.69)	
Family History	No	4.66 (0.92–26.36)	0.910 *
	Yes	4.76 (0.12–446.69)	

* Mann–Whitney U test. ** Kruskal–Wallis-H test. The groups with significant differences are labeled with letters (a) (b) (c) (d). While there is no significant difference between the groups with the same letter label, there is a significant difference (*p* < 0.05) between the groups with different letter labels. Bold values indicate statistically significant results (*p* < 0.05). Abbreviations: ACS: Acute Coronary Syndrome.

**Table 5:** Summary of correlation relationships of HALP score with other quantitative parameters in ACS patients.

Parameters	HALP Score	
Age	Rho	−0.291
	*p*	**<0.001**
WBC	Rho	0.053
	*p*	0.075
HDL	Rho	−0.055
	*p*	0.063
EF (%)	Rho	0.20
	*p*	**<0.001**
SYNTAX Score	Rho	−0.014
	*p*	0.649
TİMİ Score	Rho	−0.286
	*p*	**<0.001**
Troponin	Rho	−0.20
	*p*	**<0.001**
CRP	Rho	−0.231
	*p*	**<0.001**
D-dimer	Rho	−0.115
	*p*	**<0.001**
Ferritin	Rho	−0.239
	*p*	**<0.001**
Fibrinogen	Rho	−0.157
	*p*	**<0.001**
HEART score	Rho	−0.224
	*p*	**<0.001**
ICU time (day)	Rho	−0.143
	*p*	**<0.001**
Hospitalization time (day)	Rho	−0.054
	*p*	0.069

Spearman correlation analysis (correlation coefficient = rho). Bold values indicate statistically significant results (*p* < 0.05). Abbreviations: ACS: Acute Coronary Syndrome; WBC: White Blood Cell; EF: Ejection Fraction; CRP: C Reactive Protein; HDL: High Density Lipoprotein; ICU: Intensive Care Unit.

**Table 6:** Univariate logistic regression analysis for mortality in ACS patients.

Logistic Regression (Mortality)						
Factors	B	Nagelkerke R^2^	*p*	OR	95% GA	
					Lower Limit	Upper Limit
Age	0.056	0.068	**<0.001**	1.058	1.036	1.079
Sex	−0.006	<0.001	0.948	0.994	0.570	1.733
PIV	0.0003	0.029	**<0.001**	1.003	1.000	1.001
HALP score	−0.367	0.103	**<0.001**	0.693	0.615	0.781
Syntax Score	0.028	0.009	**0.04**	1.028	1.001	1.055
TIMI score	1.40	0.330	**<0.001**	4.055	3.048	5.396
Heart Score	1.064	0.165	**0.007**	2.897	1.338	6.273

Reference category: survival group, OR: Odd ratio. Heart score was calculated only for NSTEMI and USAP patients. Bold values indicate statistically significant results (*p* < 0.05). Abbreviations: Acute Coronary Syndrome; PIV: Pan-immun-inflammatory value.

**Table 7:** Multivariable logistic regression analysis for in-hospital mortality.

Logistic Regression (Mortality)					
Factors	B	*p*	OR	95% GA	
				Lower Limit	Upper Limit
PIV	0.0003	**<0.001**	1.003	1.000	1.001
HALP score	−0.325	**<0.001**	0.722	0.638	0.817
Syntax Score	0.02	0.154	1.020	0.993	1.048
TIMI score	1.635	**<0.001**	5.129	3.574	7.361
Heart Score	0.833	**0.038**	2.301	1.048	5.052

Reference category: survival group, OR: Odd ratio. Heart score was calculated only for NSTEMI and USAP patients. Adjusted *p* values of the multivariate models. Bold values indicate statistically significant results (*p* < 0.05). Abbreviations: PIV: Pan-immun-inflammatory value.

**Table 8:** ROC analysis of quantitative parameters for mortality in ACS patients, predictive values and cut-off values.

Parameters	AUC (%95 GA)	Cut-Off	*p*	Sensitivity (%)	Specificity (%)
PIV	0.619 (0.551–0.687)	≥1074.2	**0.001**	45.0%	79.9%
HALP score	0.722 (0.656–0.788)	≤3.58	**<0.001**	53.8%	82.0%
TIMI score	0.867 (0.831–0.903)	≥5.5	**<0.001**	93.8%	75.9%
Heart score	0.807 (0.686–0.927)	≥8.5	**<0.001**	90.8%	85.3%

AUC: Area under curve, ROC: Receiver operating characteristic, Reference Category: survival group. Heart score calculated only for NSTEMI and USAP patients. Lower values are associated with positive outcome (mortality). Bold values indicate statistically significant results (*p* < 0.05). Abbreviations: ACS: Acute Coronary Syndrome; PIV: Pan-immun-inflammatory value.

**Table 9:** Incremental prognostic value of PIV and HALP beyond TIMI score for in-hospital mortality.

Model	AUC	ΔAUC vs. TIMI	DeLong *p*-Value	Continuous NRI	IDI
TIMI score only	0.867	Reference	–	–	–
TIMI + HALP	0.903	+0.036	0.470	0.646	0.068
TIMI + PIV	0.880	+0.013	0.758	0.194	0.010

AUC values were compared using DeLong’s test. Continuous NRI and IDI were calculated to assess reclassification improvement. HALP and PIV were modeled as log-transformed continuous variables.

## Data Availability

The data that support the findings of this study are available from the corresponding author upon reasonable request.

## Abbreviations

ACS	Acute coronary syndrome
NSTEMI	Non-ST-elevation myocardial infarction
STEMI	ST-elevation myocardial infarction
PIV	Pan-Immune-Inflammatory Value
WBC	White Blood Cell
CRP	C Reactive Protein
ECG	Electrocardiography
PLT	Platelet
RDW	Red cell distribution width
LDL	Low-density lipoprotein
HDL	High-density lipoprotein
EF	Ejection Fraction
ICU	Intensive care unit
IQR	Interquartile range
SD	Standard Deviation
AUC	Area under the curve
